# Monocular inhibition reveals temporal and spatial changes in gene expression in the primary visual cortex of marmoset

**DOI:** 10.3389/fncir.2013.00043

**Published:** 2013-04-09

**Authors:** Yuki Nakagami, Akiya Watakabe, Tetsuo Yamamori

**Affiliations:** ^1^Division of Brain Biology, Department of Neurobiology, National Institute for Basic BiologyOkazaki, Japan; ^2^Department of Basic Biology, The Graduate University for Advanced Studies [SOKENDAI]Okazaki, Japan; ^3^Section of Brain Science Exploration and Training, National Institute for Physiological SciencesOkazaki, Japan

**Keywords:** activity-dependent, ocular dominance columns, cortical layer, monocular deprivation, immediate early gene, non-human primate, *in situ* hybridization

## Abstract

We investigated the time course of the expression of several activity-dependent genes evoked by visual inputs in the primary visual cortex (V1) in adult marmosets. In order to examine the rapid time course of activity-dependent gene expression, marmosets were first monocularly inactivated by tetrodotoxin (TTX), kept in darkness for two days, and then exposed to various length of light stimulation. Activity-dependent genes including *HTR1B, HTR2A*, whose activity-dependency were previously reported by us, and well-known immediate early genes (IEGs), *c-FOS, ZIF268*, and *AR*C, were examined by *in situ* hybridization. Using this system, first, we demonstrated the ocular dominance type of gene expression pattern in V1 under this condition. IEGs were expressed in columnar patterns throughout layers II–VI of all the tested monocular marmosets. Second, we showed the regulation of *HTR1B* and *HTR2A* expressions by retinal spontaneous activity, because *HTR1B* and *HTR2A* mRNA expressions sustained a certain level regardless of visual stimulation and were inhibited by a blockade of the retinal activity with TTX. Third, IEGs dynamically changed its laminar distribution from half an hour to several hours upon a stimulus onset with the unique time course for each gene. The expression patterns of these genes were different in neurons of each layer as well. These results suggest that the regulation of each neuron in the primary visual cortex of marmosets is subjected to different regulation upon the change of activities from retina. It should be related to a highly differentiated laminar structure of marmoset visual systems, reflecting the functions of the activity-dependent gene expression in marmoset V1.

## Introduction

The primary visual cortex (V1) of primates is estimated to occupy more than 30% of the cerebral cortex (Collins et al., 2012). It has many characteristic features that enable highly complex informational processing, e.g., formation of distinct functional columnar structures (ocular dominance and orientation columns, or color domains, etc.) (Hubel and Wiesel, 1977; Livingstone and Hubel, 1984), or parallel processing (Joels and de Kloet, 1992; Nassi and Callaway, 2009). We previously showed that the expression of a group of genes, such as *OCC1* (*FSTL1*), *5-hydroxytryptamine* (serotonin) *receptor 1B* and *2A* (*HTR1B* and *HTR2A*, respectively), *Testican-1*, and *Testican-2*, are highly enriched in the thalamorecipient layers of V1 of adult macaques (Tochitani et al., 2001; Takahata et al., 2009; Watakabe et al., 2009). Their V1-enriched expression patterns were conserved in several species of primate but not in ferrets and mice (Takahata et al., 2008, 2012), suggesting that there are primate-specific mechanisms for expression of these genes. An important common feature to these genes was the activity-dependent expression in V1, which we showed by monocular inactivation of retinal activity using tetrodotoxin (TTX) (Tochitani et al., 2001; Takahata et al., 2009; Watakabe et al., 2009; Yamamori, 2011). Whereas this experiment revealed the requirement for retinal activity in gene expression in V1, it has not been clear how the incoming visual inputs induce the expression of these genes.

Synaptic transmission triggers the expression of a group of genes, which play roles in neural plasticity, differentiation, proliferation etc. (Flavell and Greenberg, 2008; Fowler et al., 2011). Among these genes, immediate early genes (IEGs) including *c-Fos* and *Zif268* are defined as the genes that are rapidly and transiently expressed within minutes to several hours from stimulus onset (Morgan et al., 1987; Sheng and Greenberg, 1990), and have been used as the markers for neural activities after sensory stimulation. Studies for how visual stimulation causes the induction of IEGs in the visual cortex have been also done in various mammalian species (Worley et al., 1991; Rosen et al., 1992; Chaudhuri and Cynader, 1993; Montero and Jian, 1995; Kaplan et al., 1996; Markstahler et al., 1998; Arckens et al., 2000; Soares et al., 2005; Warner et al., 2012). Previous studies in rodents demonstrated that visual stimulation induces the expressions of Zif268 and c-Fos proteins at the peak level within 1 h from stimulus onset (Worley et al., 1991; Zangenehpour and Chaudhuri, 2002), suggesting that input-driven gene activation in V1 reaches the maximum level within a short period of time. To our knowledge, however, there has been no information about visually evoked transcription in primate V1 during the early time course within 1 h. Here, we designed a series of monocular visual stimulation experiments using adult marmosets, in order to dissect underlying molecular mechanisms upon changes of visual inputs in primates.

For our experiments, we selected common marmosets (*Callithrix jacchus*), a New World monkey, because of its size, ease of handling, and transgenic (Sasaki et al., 2009) and gene manipulation potentials (e.g., Watakabe et al., 2012). In the marmoset vision research, there have been debates whether ocular dominance columns (ODCs) exist (Sengpiel et al., 1996; Markstahler et al., 1998; Chappert-Piquemal et al., 2001; Roe et al., 2005) or not exist (Spatz, 1979, 1989; McLoughlin and Schiessl, 2006; Valverde Salzmann et al., 2012) in adult marmosets. With particular relevance to our study, Markstahler et al. (1998) reported columnar ZIF268 immunostaining in layer IVCβ 2 h after monocular visual stimulation following transient (24 h) monocular TTX injection, which they called “physiological ODCs.” To investigate the visually evoked gene expression in primates, marmoset V1 is potentially a very good model.

In the present study, we modified the Markstahler's method (1998) to examine the mRNA expression of a set of activity-dependent genes including *c-FOS, ZIF268*, and *ARC* in adult marmoset V1. Using this approach first, we demonstrated strong evidence for the segregation of right and left eye inputs in marmoset V1. Second, we have found that spontaneous activity has a critical role in the expression of *HTR1B* and *HTR2A* mRNAs in these primate-specific domains in an activity-dependent manner (Watakabe et al., 2009; Takahata et al., 2012). Last, we found that each of these activity-dependent genes revealed a different spatial and temporal time course upon visual stimulation. These results suggest that the regulation of each neuron in marmoset V1 is subjected to different regulation upon the change of activities from retina.

## Materials and methods

### Ethics statement

All the experiments were conducted in accordance with the guidelines of the National Institutes of Health, and the Ministry of Education, Culture, Sports, Science and Technology (MEXT) of Japan, and were approved by the Animal Care and Use Committee in the National Institutes of Natural Sciences. We made all efforts to minimize the number of animals used and their suffering.

### Experimental animals and visual manipulation procedure

**Figure 1:**
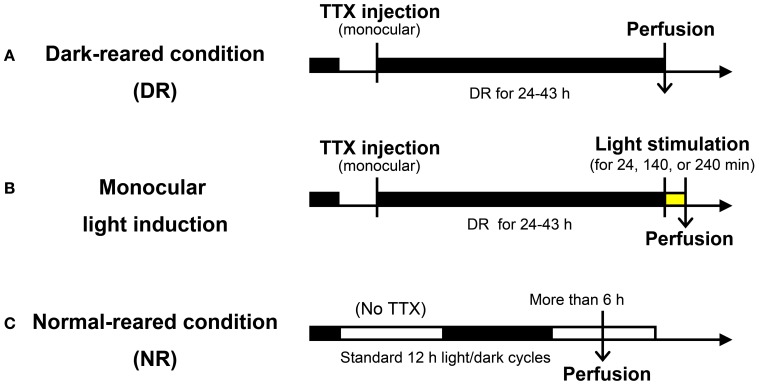
**Experimental procedure used for light induction experiments. (A)** Dark-reared (DR) condition: following TTX injection into one eye, marmosets were kept in the dark for 24–43 h before sacrifice. **(B)** Monocular light induction: following the same experimental procedure in **(A)**, light stimuli were given for 24, 140, or 240 min before sacrifice. **(C)** Normal-reared (NR) condition: marmosets were housed under standard 12 h light/dark cycles without TTX injection. Perfusion was done at the time when animals were under the light cycle more than 6 h. Detailed experimental conditions are written in the text and “Materials and Methods.”

A total of 16 adult common marmosets (*Callithrix jacchus*, 20–98 months, either sex, weighing 257–472 g) were used during the course of this study (11 marmosets among them were also used for other studies as well). These marmosets were kept under standard 12 h light: 12 h dark condition, until the manipulation was started. As shown in Figure 1, the visual manipulation typically started from the dark-reared (DR) condition, during which the marmosets were deprived of any possible light source. To achieve the DR condition, in addition to turning the room light off, we carefully covered the cage with a lightproof shield.

Five marmosets were visually intact and did not receive TTX injection. Three of them were used as the normal-reared (NR) condition marmosets: these marmosets were kept under 12 h light: 12 h dark condition and sacrificed more than 6 h after the light cycle had started. Two of them were kept under the DR condition for one night and were exposed to a bright light from the front side of their cages for 2 h before sacrifice (NR 2h; control of TTX injection). The illuminance was 300 and 1500 lx under the NR condition and light stimulation, respectively. There was no object to hide from light sources in their cages.

Eleven other marmosets received monocular intravitreous injection of TTX to block retinal impulse activity. Under anesthesia of the ketamine and xylazine mixture (25 and 2.0 mg/kg, respectively), 1.4–1.6 μl of TTX (4.7 mM; Wako Pure Chemical Industries, Ltd., Osaka, Japan) was manually injected into the vitreous cavity of one eye using a Hamilton syringe. After injection, loss of the pupillary reflex in the injected eye was confirmed. After awakening from anesthesia, to completely reduce the transcription products already induced by normal breeding to basal level, we then kept the marmosets under the DR condition for 24–43 h prior to stimulation. Light stimulation was done by exposing to a bright light from the front side of the cages for 0, 24, 140, and 240 min before being anesthetized for sacrifice. Information of the experimental procedures for each animal is summarized in Table 1.

**Table 1:** **Marmosets used for monocular visual stimulation experiments**.

**ID**	**Sex**	**Age (month)**	**BW (g)**	TTX^*^ **(μl)**	DR^**^ **(h)**	**Visual stimuli (min)**
NR_1	Female	22	337	–	(12)	(NR)
NR_2	Female	22	471.9	–	(12)	(NR)
NR_3	Male	26	302	–	(12)	(NR)
NR 2 h_1	Male	23	421	–	12	130
NR 2 h_2	Male	24	317	–	13	120
DR_1	Male	21	301	L-1.5	25	0
DR_2	Female	25	346	R-1.5	43	0
DR_3	Female	47	345.7	L-1.5	41.5	0
MD 24 min_1	Female	73	307.8	L-1.4	24.5	22.5
MD 24 min_2	Female	79	354.2	L-1.6	39	23.5
MD 24 min_3	Female	42	463	L-1.6	40	24
MD 140 min_1	Male	20	320	L-1.4	24	135
MD 140 min_2	Female	98	334	L-1.5	24	145
MD 240 min_1	Male	25	257	L-1.6	43	240
MD 240 min_2	Male	25	334	L-1.5	43	240
MD 240 min_3	Female	69	465	L-1.4	42	238

* L, left eye; R, right eye; –, no treatment.

** Marmosets were kept under the DR condition for the indicated length of time prior to stimulation. NR marmosets were reared under standard 12 h light: 12 h dark condition. See “Materials and Methods” for details.

### Tissue preparation

The animals were sacrificed with overdose of sodium pentobarbital (100 mg/kg, intraperitoneally) following anesthesia of a mixture of ketamine and xylazine (30 and 2.7 mg/kg body weight, respectively; intramuscularly). Before the injection of sodium pentobarbital, the loss of the pupillary reflex in the TTX-injected eye was confirmed in all marmosets except the DR animals. After confirming that the marmosets were in deep anesthesia, they were perfused transcardially with 0.9% NaCl containing 2 U/mL heparin, followed by fixation with 4% paraformaldehyde (PFA) in 0.1 M phosphate buffer, pH 7.4. We carefully minimized the time period between anesthesia and infusion of PFA, particularly for the cases of short-term light induction. For example, in the case of 24-min-light stimulation, the marmosets started to receive 4% PFA infusion within 35 min from the onset of light exposure. For the three marmosets with no visual stimulation (Table 1, DR_1,2,3), the anesthesia was induced in the dark room using night vision goggles, and the perfusion was performed under low light until PFA infusion started.

The brains were postfixed for 5 h at room temperature and then cryoprotected with 30% sucrose in 0.1 M phosphate buffer at 4°C. The tissue sections were made on a freezing microtome (20 μm thickness for IHC and double ISH; 20 to 25 μm thickness for single ISH).

### Synthesis of RNA probes

The probes used for ISH experiments were PCR-cloned by using the primers listed in Table 2. These primers were designed based on the marmoset sequences, deposited on Ensemble (http://www.ensembl.org) (Flicek et al., 2012). The cDNA fragments were obtained by RT-PCR using the total RNA purified from the occipital part of the marmoset neocortex. After subcloning, the sequences were checked using the BLAT alignment tool (Kent, 2002) for the marmoset in the UCSC Genome Browser database (http://genome.ucsc.edu/) (Kent et al., 2002). The digoxygenin (DIG)- and fluorescein isothiocyanate (FITC)-labeled riboprobes were produced using these plasmids as templates for *in vitro* transcription. For the detection of *ZIF268, HTR1B, HTR2A*, and *VGluT1* mRNAs, we used the probes previously used for monkey ISH (Komatsu et al., 2005; Takahata et al., 2008; Watakabe et al., 2009). We also conducted ISH for the sense probes of each gene to confirm the specificity of the antisense probes (data not shown).

**Table 2:** **ISH probes used in current study**.

**Probe name**	Species^*2^	Reference^*3^	**Primer**	**Forward (5′ → 3′)**	**Length**
				**Reverse (3′ → 5′)**	
zif268-1	Rhesus monkey	NM_001964	GCACCCACACAGGCGAAAAG		352
			GCAGGGGGAACAGAGGAGTA		
zif268-2^*1a^	Rhesus monkey	NM_001964	CCCAGGACAATTGAAATTTGCT		798
			AAGGCACCAAGACGTGAAAC		
ARC-1	Marmoset	ENSCJAT00000023550	ATCCTGCAGATCGGGAAGTG		343
			CACTGCCCACCGGGTACTTG		
ARC-2	Marmoset	ENSCJAT00000023550	CAGGAGCCAGCCGAGGCCCA		522
			CAGGTCGTCTTGCACCTCCA		
c-FOS	Marmoset	ENSCJAT00000040535	GCAGACCGAGATTGCCAACC		607
			TCACAGGGCCAGCAGTGTGG		
5-HT1B	Marmoset	ENSCJAT00000043359	TCCTCTACACGGTCTACTCC		980
			CAAGTACTGCCAGGCTGTATGT		
RC15i^*1b^	African green	Genbank No. AL049595			1551
(5-HT1B)	monkey				
5HT2AR-1^*1b^	Rhesus monkey	NM_000621	GCTCAACTACGAACTCCCTAAT		766
			AGTAGCTTCTTTCTGGAGTGAC		
5HT2AR-2^*1b^	Rhesus monkey	NM_000621	CCTTGTCATGCCCGTGTCCA		833
			TTYTCCTTGTACTGRCACTG		
VGluT1-1^*1c^	Rhesus monkey	NM_020309	CCGCTACATTATCGCCATCA		892
			CGATGGGCACGATGATGGCT		
VGluT1-2^*1c^	Rhesus monkey	NM_020309	TGCGCAAGTTGATGAACTGC		834
			CCTGAAAGGAGAGATTTGAAAC		
VGluT1-3^*1c^	Rhesus monkey	NM_020309	TTGTGGTTTTGAGGCACCCA		760
			CAGTCACAGAGACAGAGACAC		

*1 : ISH probes used in previous works (a, Takahata et al., 2008; b, Watakabe et al., 2009; c, Komatsu et al., 2005).

*2 : Species of the cDNA cloned in each probe.

*3 : Reference sequences of marmoset, macaque, or human used to design PCR primers.

### *In situ* hybridization

Single-colored ISH was carried out as described previously with minor modifications (Liang et al., 2000; Komatsu et al., 2005). Free-floating sections were treated with 5 μg/mL proteinase K for 30 min at 37°C. After acetylation, the sections were incubated in a hybridization buffer [5× standard saline citrate (SSC), 2% blocking reagent (Roche Diagnostics, Basel, Switzerland), 50% formamide, 0.1% N-lauroylsarcosine (NLS), 0.1% SDS] containing 0.5 μg/mL DIG-labeled riboprobes at 60–65°C. Hybridized sections were washed twice in 2× SSC/50% formamide/0.1% NLS for 20 min at the same temperature for hybridization, then treated at 37°C in RNase buffer [10 mM Tris-HCl, pH 8.0, 1 mM ethylenediaminetetraacetic acid (EDTA), 500 mM NaCl] containing 20 μg/mL RNase A (Sigma-Aldrich, St. Louis, MO) for 30 min. The sections were further washed twice at 37°C in 2× SSC/0.1% NLS, and then twice in 0.2× SSC/0.1% NLS. Hybridization signals were visualized by alkaline phosphatase immunohistochemistry followed by nitro-blue tetrazolium/5-bromo-4-chloro-3-indolylphosphate (NBT/BCIP) detection (Roche Diagnostics, Tokyo, Japan).

Fluorescence double-colored ISH was performed using DIG- and FITC-labeled riboprobes as described previously (Komatsu et al., 2005; Watakabe et al., 2010). The hybridization was carried out as described above, except that both DIG- and FITC-labeled riboprobes were used for the hybridization. After blocking in 1% blocking buffer (Roche Diagnostics) for 1 h, DIG- and FITC-labeled riboprobes were detected in two different ways. For the detection of the FITC probes, the sections were incubated with an anti-FITC antibody conjugated with horseradish peroxidase (1:5000 in the blocking buffer; Jackson ImmunoResearch Laboratories, West Grove, PA; #200-032-037) for 2–4 h at room temperature. After washing in TNT (0.1 M Tris-HCl, pH 7.5, 0.15 M NaCl, 0.1% Tween 20) three times for 15 min, the sections were treated with 1:100-diluted TSA-Plus reagents for 30 min in accordance with the manufacturer's instruction (Perkin-Elmer, Wellesley, MA), and the FITC signals were converted to dinitrophenyl (DNP) signals. After washing in TNT three times for 10 min, the sections were incubated for 2–4 h at room temperature or overnight at 4°C with an anti-DNP antibody conjugated with Alexa 488 [1:500, Molecular Probes (Life Technologies Corporation), Carlsbad, CA] in 1% blocking buffer for the fluorescence detection of the DNP signals. At this point, an anti-DIG antibody conjugated with alkaline phosphatase (1:1000, Roche Diagnostics) was included in the incubation, for the detection of the DIG probes. The sections were washed thrice in TNT, once in TS 8.0 (0.1 M Tris-HCl, pH 8.0, 0.1 M NaCl, 50 mM MgCl_2_), and the alkaline phosphatase activity was detected using an HNPP fluorescence detection set (Roche Diagnostics) in accordance with the manufacturer's instruction. The incubation for this substrate was carried out for 40 min and stopped by washing in phosphate buffered saline (PBS) containing 0.5 mM EDTA. The sections were then counterstained with Hoechst 30442 (Molecular Probes) diluted in PBS to 1:1000 for 5 min.

### Immunohistochemistry

We used anti-c-Fos polyclonal rabbit IgG antibody (Santa Cruz Biotechnology, Santa Cruz, CA; sc-52), which was raised against a peptide mapping at the N-terminus of c-FOS of human origin, for c-Fos immunoreactivity. We previously confirmed the specificity and used this antibody in rats (Sakata et al., 2002; Hirokawa et al., 2008). For marmoset c-FOS IHC, the monocularly deprived and normal columns in V1 showed clear contrast between two types of columns as shown in the result figures, which confirmed the specificity as internal controls.

For immunoperoxidase reaction, the free-floating sections were incubated in Tris-buffered saline (TBS) containing 1% H_2_O_2_ for 30 min at room temperature. After rinsing in TBS, the sections were immersed for 1 h at room temperature in the blocking buffer (5% bovine serum albumin, 0.1% Triton X-100, and 4% normal goat serum in TBS). Reaction with an anti-c-Fos antibody (1:1200) was performed in the blocking buffer overnight at 4°C. Following incubation with an biotinylated donkey anti-rabbit IgG (1:4000; Jackson ImmunoResearch Laboratories) in the blocking buffer for 2 h at room temperature, the sections were processed with an avidin-biotinylated horseradish peroxidase complex (1:200; Vectastain ABC Elite kit, Vector Laboratories, Burlingame, CA) in TBS at room temperature for 1 h and the immunoreaction was visualized by staining with nickel-enhanced coloring solution [0.2 mg/mL diaminobenzidine (DAB), 0.03% H_2_O_2_, 0.03% nickel chloride in TBS].

### Data analysis

In this study, we adopted Brodmann's nomenclature for the V1 layering. We determined the layer positions based on Nissl staining of the adjacent section. To identify the precise lamina positions, we also performed double ISH of the genes of interest with several layer marker genes, such as *Nurr1* (*Nr4a2*), *Neurofilament* (*NEFM*), *ER81* (*ETV1*), and *VGluT1* (Watakabe et al., 2007). In this paper, we designated the ODCs as the active-eye and the inactive-eye columns that received projection from the intact and the TTX-injected eyes, respectively.

The images for ISH and IHC were obtained using a digital color camera DP70 (Olympus, Tokyo, Japan) attached to a BX-51 microscope (Olympus). All the figures obtained in the ISH and the IHC experiments were adjusted for appropriate contrast using Adobe Photoshop (Adobe Systems, San Jose, CA). Although the sections for ISH shrank, the scale bars in the figures show the size of mounted sections, which were not adjusted for shrinkage.

The laminar profiles shown in Figures 3, 6 were quantified by measuring the integrated optical densities (IODs) of the ISH signals in the designated layers as follows. First, we obtained columnar images of active-eye and inactive-eye columns, which contained from layer I to WM vertically, and pasted all images measuring IODs in one canvas for each gene using Adobe Photoshop. We obtained the layer values for an active-eye column and for an inactive-eye column per section. To obtain the average signal intensity, five or six sections from two to three different marmosets in each time point (Table 1). Second, two vertical lines were set within the active- or inactive-eye column to obtain the line profile using the “line profile (thick vert)” menu of Image-Pro Plus. In this menu, the 8-bit gray-scale values of the pixels at each vertical position were averaged to determine the optical density at that position. Third, this line profile was referenced to the cortical layers determined as described above. Fourth, a portion (30 pixel height) of each layer (or sublayer) was set as the ROI for IOD measurement. That is, the OD values at particular lamina positions were integrated as the “signal intensity” of this layer. Signal to noise ratio of ISH signal was high enough so that the background level can be negligible. In order to combine data from multiple samples we carefully perform the tissue processing (e.g., storage of samples, pegged coloring time) and carried out ISH of cortical tissues of different animals at the same condition. Within a range of difference in the length of the dark-rearing time in the current study, the expression profiles were confirmed to be similar among the samples obtained at the same time point.

## Results

### Functional ODCs in V1 of adult marmosets throughout layers II–VI visualized by monocular light induction experiment

To investigate the time course of activity-dependent gene expression in V1, we carried out a series of light induction experiments in monocularly inactivated marmosets. As shown in Figure 1, following intravitreous injection of TTX to monocularly block retinal activity, marmosets were kept under the DR condition for one or two days to reduce the level of activity-dependent gene expression to the basal level. We then exposed them to a bright light from the front side of the cages for different time periods before sacrifice. In this way, we were able to objectively compare the expression patterns between active- and inactive-eye columns.

**Figure 2:**
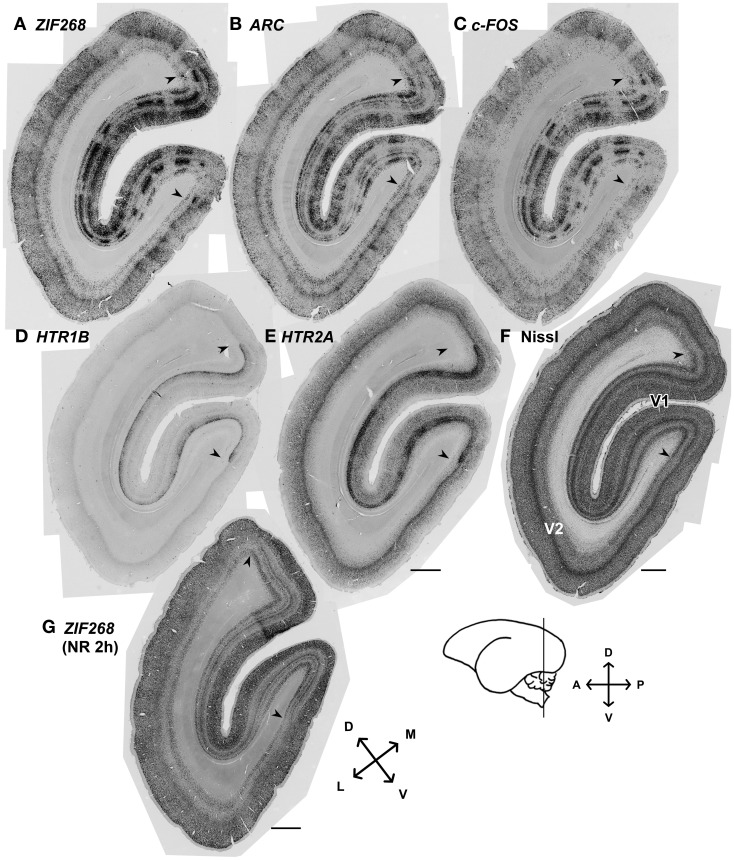
**mRNA expression patterns of five activity-dependent genes induced by monocular light stimulation in visual cortices of adult marmosets. (A–E)** mRNA expression patterns for activity-dependent genes we examined in the visual cortex 24 min after light stimulation: **(A)** *ZIF268*, **(B)** *ARC*, **(C)** *c-FOS*, **(D)** *HTR1B*, and **(E)** *HTR2A*. Columnar patterns within V1 indicate ocular dominance columns. **(F)** Cortical section stained for Nissl substance. Adjacent sections were analyzed in **(A–F)**. Magnified images at other time points were shown in Figures 3, 5. **(G)** The mRNA expression pattern for *ZIF268* in the cortical section obtained from a marmoset which was received 2 h-light stimulation without monocular TTX injection (NR 2h). No stripe pattern was observed in any layers of V1. Arrowheads indicate V1/V2 boundaries. Cortical sections were prepared from the position depicted by the line on the brain diagram. D, dorsal; V, ventral; A, anterior; P, posterior; L, lateral; and M, median. Scale bars: 1 mm.

Although there had been controversy with regard to the presence of ODCs in marmosets, we always observed stripe patterns of expression for the activity-dependent genes as previously observed in macaques (Takahata et al., 2009; Watakabe et al., 2009) (Figure 2). Consistent with the previous report (Markstahler et al., 1998), the active-eye columns were visualized by ISH for *ZIF268* in the marmoset visual cortex (Figure 2A). Similar stripe patterns were observed for two other IEGs, *ARC* and *c-FOS*, as well (Figures 2B,C). In this series of experiments, all monocularly inactivated marmosets showed clear stripe signals of IEGs through layers II–VI without exception. The distinction between active- and inactive-eye columns was less conspicuous in layers outside layer IVCβ, suggesting relatively less segregation of inputs in other layers. We also confirmed that *HTR1B* and *HTR2A* mRNAs exhibit stripe patterns in layer IVC in these monocularly inactivated marmosets (Figures 2D,E). Figure 2G shows the ISH of *ZIF268* in the cortical section obtained from a marmoset which was received 2 h-light stimulation without monocular TTX injection (NR 2 h). Note that there is no stripe pattern in any layers in V1 (Figure 2G). These findings demonstrate the clear segregation of ODCs in adult marmoset V1.

### Differential requirements for visual inputs in the inductions of *HTR1B* and *HTR2A* genes

**Figure 3:**
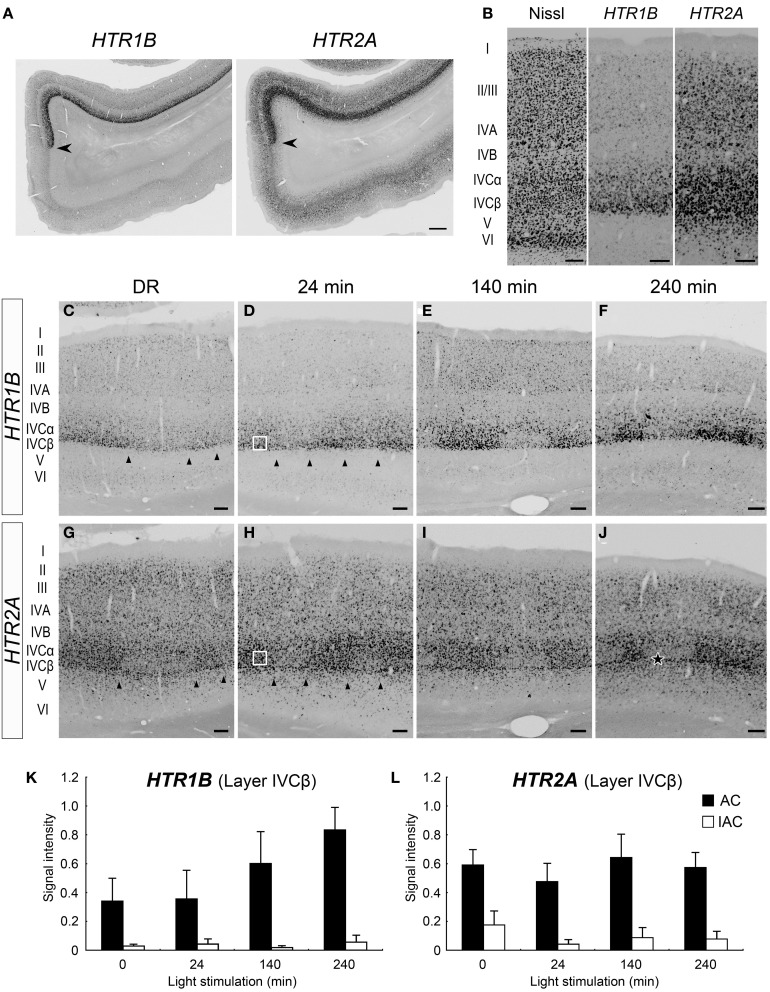
**Expressions of *HTR1B* and *HTR2A* mRNAs in marmoset V1. (A)** mRNA expressions of *HTR1B* and *HTR2A* in the visual cortex of the normal-reared (NR) marmoset as in Figure 1C. Arrowheads indicate the V1/V2 boundaries. Note the area-specific expression pattern of *HTR1B* and *HTR2A* mRNAs in V1. Scale bar: 500 μm. **(B)** Laminar distributions of *HTR1B* and *HTR2A* mRNAs in V1. Both genes were higher expressed in layer IVC. **(C–J)** ISH signals of *HTR1B* **(C–F)** and *HTR2A* **(G–J)** mRNAs in V1 of monocular inactivated marmosets. Twenty-four minutes **(D,H)**, 140 min **(E,I)**, and 240 min **(F,J)** after monocular light induction as in Figure 1B. **(C,G)** Dark-reared animals (DR) as in Figure 1A. The filled star in panel **(J)** indicates the position of sublayer where *HTR2A* mRNA was constantly expressed across ODCs. Arrowheads indicate boundaries of ODCs, which were demarcated on the basis of the pattern of *ZIF268* mRNA in the adjacent sections. Open squares in panels **(D,H)** indicate the regions of layer IVCβ isolated for quantification analysis as examples. The regions of other time points are also taken at similar layer positions for quantification analysis in **(K)** and **(L)**. Scale bars: 100 μm. **(K,L)** Quantification of mRNA signal levels of *HTR1B* **(K)** and *HTR2A* **(L)** in layer IVCβ of V1 for each time point. Filled bars, active-eye columns (AC); open bars, inactive-eye columns (IAC). For this calculation, five sections (for DR and 140 min) and six sections (for 24 and 240 min) were obtained from two or three marmosets in each time point (see Table 1). Error bars show SD.

As previously reported (Takahata et al., 2012), both *HTR1B* and *HTR2A* mRNAs were preferentially expressed in marmoset V1 (Figure 3A). The expression of *HTR1B* mRNA was mostly confined to layer IVC (particularly strong in layer IVCβ) and weakly observed in layers II, III, and IVA (Figure 3B). *HTR2A* mRNA was expressed in layers II–V, with higher expression in layer IVC (Figure 3B). Next, we examined the time course of *HTR1B* and *HTR2A* gene expressions in response to the visual stimulation. In regard to *HTR1B* and *HTR2A*, the effect of monocular inactivation by TTX was observed in layer IVC. *HTR1B* mRNA was expressed at a low level under the DR condition and gradually increased its expression level in proportion to the length of visual stimuli (Figures 3C–F). As quantified in Figure 3K, although the *HTR1B* mRNA level did not significantly change during the first 24 min, it increased from 140 to 240 min. The expression levels of *HTR2A* mRNA, on the other hand, were similar within the stimulus conditions including the DR (Figures 3G–J). In our experiments, we did not find any significant increase in signal intensities for *HTR2A* mRNA, during visual stimulation within 240 min (Figure 3L), indicating that *HTR2A* mRNA was expressed at a certain level without light stimulation. Note that blocking the retinal activity by TTX reduced the level of both *HTR1B* and *HTR2A* mRNAs even under the DR condition (Figures 3C,G). This observation suggested that spontaneous retinal activity sustained a level of *HTR1B* and *HTR2A* mRNA expression, irrespective of light stimulation. Thus, a series of monocular light induction experiment performed here revealed: first, the difference of *HTR1B* and *HTR2A* mRNA expressions in terms of their requirement for visual inputs to achieve the maximum level of expression, and second, the requirement for spontaneous retinal activities in their mRNA expression.

**Figure 4:**
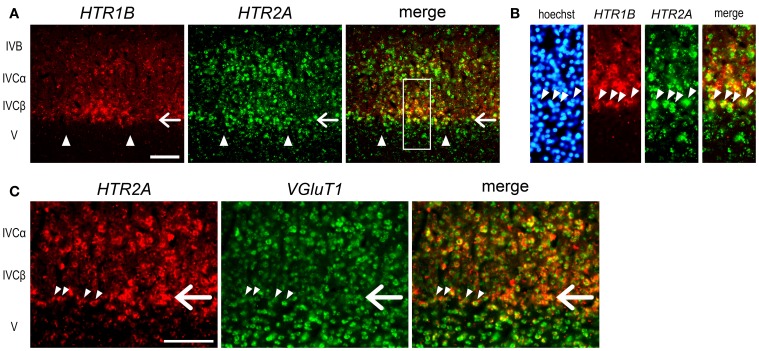
**Double ISH of *HTR1B* and *HTR2A* in V1 of monocularly stimulated marmosets. (A)** Double ISH of *HTR1B* (red) and *HTR2A* (green) mRNAs 240 min after visual stimulation. *HTR1B* and *HTR2A* mRNAs were coexpressed in most of the same neurons in active-eye columns of layer IVC in V1. White arrows indicate the sublayer where *HTR2A* mRNA was constantly expressed regardless retinal activity. This is the same sublayer which is indicated by a filled asterisk in Figure 3J. Arrowheads indicate the boundaries of ODCs. **(B)** Magnified images of the region indicated by open box in **(A)**. Arrowheads indicate the neurons of the *HTR2A*-positive sublayer. These neurons correspond to the lower end of *HTR1B*-positive neurons, suggesting that this sublayer is a part of layer IV and that there are difference in activity-dependency of mRNA expression between *HTR1B* and *HTR2A*. **(C)** Double ISH of *HTR2A* (red) and *VGluT1* (green) mRNAs 140 min after visual stimulation. White arrows indicate the similar sublayer in **(A)**. Arrowheads indicate the neurons in this sublayer. *VGluT1* mRNA was also expressed in these neurons. Scale bars: 100 μm.

The expression profiles of *HTR1B* and *HTR2A* mRNAs also revealed a characteristic sublamina configuration of marmoset V1, which may be difficult to see by other methods. At the border between layers IVCβ and V, we were consistently able to observe a narrow band of *HTR2A* mRNA that was present across the active- and inactive-eye columns (filled star, Figure 3J and at a similar position through the panels of **G–I**). To examine whether this sublayer belongs to layer IVC or V, we performed the double ISH of *HTR1B* and *HTR2A*. White arrows in Figure 4A indicate the sublayer where *HTR2A* mRNA was constantly expressed across ODCs. In magnified images of Figure 4B, arrowheads indicate the neurons in this sublayer, and *HTR1B* mRNA coexpressed in these neurons, suggesting this sublayer is a part of layer IVC. Although these *HTR2A*-positive sublayer neurons were excitatory neurons (white arrow, Figure 4C), the expression of *HTR2A* mRNA was not activity-dependent. These results indicate the different regulation of gene expression between *HTR1B* and *HTR2A* genes in response to retinal activity in this sublayer.

### Different time course of IEG expression in layers of V1

**Figure 5:**
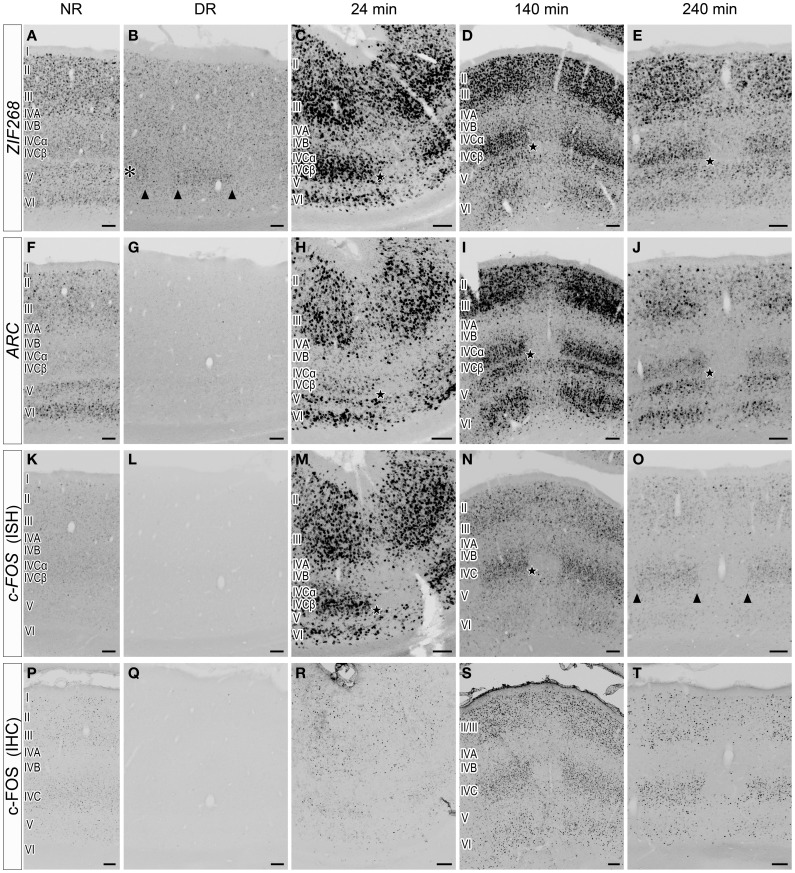
**Laminar distribution of IEG mRNA expression in V1 induced by monocular light stimulation. (A–O)** Expressions of *ZIF268* **(A–E)**, *ARC* **(F–J)**, and *c-FOS* **(K–O)** mRNAs in V1. Twenty-four minutes **(C,H,M)**, 140 min **(D,I,N)**, and 240 min **(E,J,O)** after monocular light induction as in Figure 1B. **(B,G,L)** Dark-reared animals (DR) as in Figure 1A. **(A,F,K)** Normal-reared animals (NR) as in Figure 1C. Adjacent sections were analyzed by the probe of the indicated gene under each condition. The asterisk in panel **(B)** indicates layer IVCβ. The arrowheads in panels **(B,O)** indicate the boundaries of ODCs. Filled stars indicate the positions of a narrow sublayer within layer IVC **(C–E,H–J,M,N)**; this sublayer was examined by a double ISH in Figure 8 (shown by white arrows). Scale bars: 100 μm. See also Figure 6, in which the mRNA level of each gene was quantified in each layer. **(P–T)** c-FOS immunostaining in V1. The sections adjacent to those in panels **(K–O)** were used. Scale bars: 100 μm.

The slow time course of *HTR1B* induction led us to examine a faster time course of the mRNA expression of *c-FOS, ARC*, and *ZIF268* (Flavell and Greenberg, 2008; Fowler et al., 2011). Figure 5 shows laminar distributions of the mRNA expression of these IEGs. Under the DR condition, *ZIF268* mRNA was expressed at a very low level in layer IVCβ of active-eye columns (Figure 5B), suggesting that spontaneous inputs from normal retina induce *ZIF268* mRNA expression in layer IVCβ of V1. *ARC* and *c-FOS* mRNAs were totally absent under the DR condition (Figures 5G,L), indicating that visual stimulation is required to activate the expression of these two IEGs.

**Figure 6:**
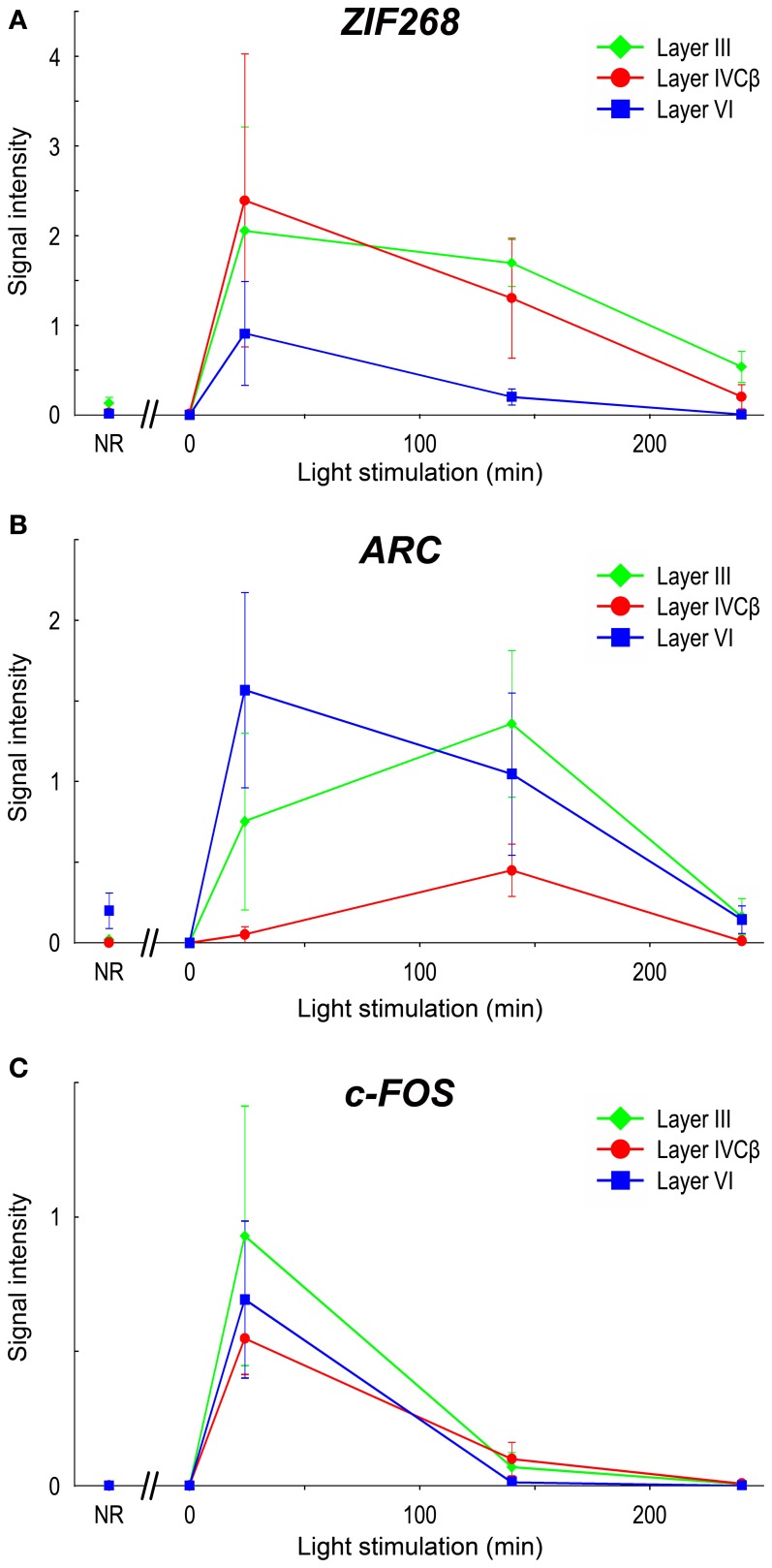
**mRNA levels of IEGs in marmoset V1 induced by monocular light stimulation.** Signal intensities of ISH of IEGs in layers III, IVCβ, and VI in V1 are shown: **(A)** *ZIF268*, **(B)** *ARC*, and **(C)** *c-FOS*. To quantify laminar distribution, signal intensities of ISH were calculated under each stimulus time in each layer (or sublayer) (see “Materials and Methods” for details). NR: mRNA levels in V1 of NR marmosets. For this calculation, five or six sections were obtained from two or three marmosets in each time point (see Table 1). The plots for different layers are indicated by colors (green: layer III; red: layer IVCβ; blue: layer VI). Error bars show SD.

Upon light stimulation, the expression of all the IEGs was rapidly induced across layers in V1. Interestingly, the time course of expression was quite different among layers, especially in layers III, IVC, and VI, signal intensities of ISH were rapidly changed within short term (quantified data in Figure 6). For example, *ARC* mRNA was abundantly expressed in layers II, III, V, and VI within 24 min, but very faint in layer IVC at this time point (about 10% against its peak we examined) (Figure 5H). After 140 min, *ARC* mRNA expression was observed in layers II, III, IVC, V, and VI at the peak of the expression (Figure 5I). After 240 min the expression level of *ARC* mRNA decreased in all layers (2.4–12% against its peak of each layer), particularly pronounced in the upper layers (Figure 5J). As shown in Figure 6B, the peak of *ARC* mRNA expression in layers III and IVC was at 140 min, whereas that in layer VI was at 24 min. Under the NR condition (i.e., marmosets exposed to light over 6 h and without any TTX injection), the signals of *ARC* mRNA were observed in layers II, III, V, and VI, but not in layer IVC (Figure 5F).

In the case of *c-FOS*, its mRNA was expressed in layers II, III, IVA, IVC, V, and VI within 24 min and then rapidly decreased and almost disappeared after 240 min (Figures 5M–O). The expression level of *c-FOS* mRNA was strongest at 24 min among the time points examined and rapidly decreased less than 1.8–18% against its peaks in all layers within 140 min (Figure 6C). Note that mRNA expression in layer VI had almost gone within 140 min even though remained in other layers (Figure 5N), suggesting that the reduction of mRNA was also differently regulated in each layer. *c-FOS* mRNA was barely observed under the NR condition in all layers (Figure 5K).

Although the laminar distribution of *ZIF268* mRNA was similar to that of *c-FOS* mRNA (Figures 5C–E), the time course of *ZIF268* mRNA expression was different. As shown in Figure 6A, the mRNA expression of *ZIF268* was rapidly induced in all layers at 24 min at the peak level. *ZIF268* mRNA sustained relatively high expression level at 140 min (82.5, 54.5, and 22% against its peak levels in layers III, IVCβ, and VI, respectively). A certain level of mRNA expression was observed in layers II–VI even under the NR condition (Figure 5A). In contrast to *ARC* mRNA, *ZIF268* mRNA was expressed stronger in the upper layers under the NR condition.

The time course of expression is different for mRNA and protein (Zangenehpour and Chaudhuri, 2002; Kovacs, 2008). Using the anti-c-FOS antibody, we compared the difference between mRNA and protein expressions. At 24 min, c-FOS immunoreactivity was sparsely observed in layer II/III, in contrast to strong expression of *c-FOS* mRNA at the same time point (Figures 5M,R). The c-FOS immunostaining signal became abundantly observed at 140 min (Figure 5S) and was still present at 240 min, at which point *c-FOS* mRNA was almost gone (Figures 5O,T). Under the NR condition, however, c-FOS immunostaining signals became very weak (Figure 5P), indicating that the protein expression followed mRNA expression with a certain time lag in the same cells in marmoset V1.

### Heterogeneity of activity-dependent regulations simultaneously occurred in the same neuron

**Figure 7:**
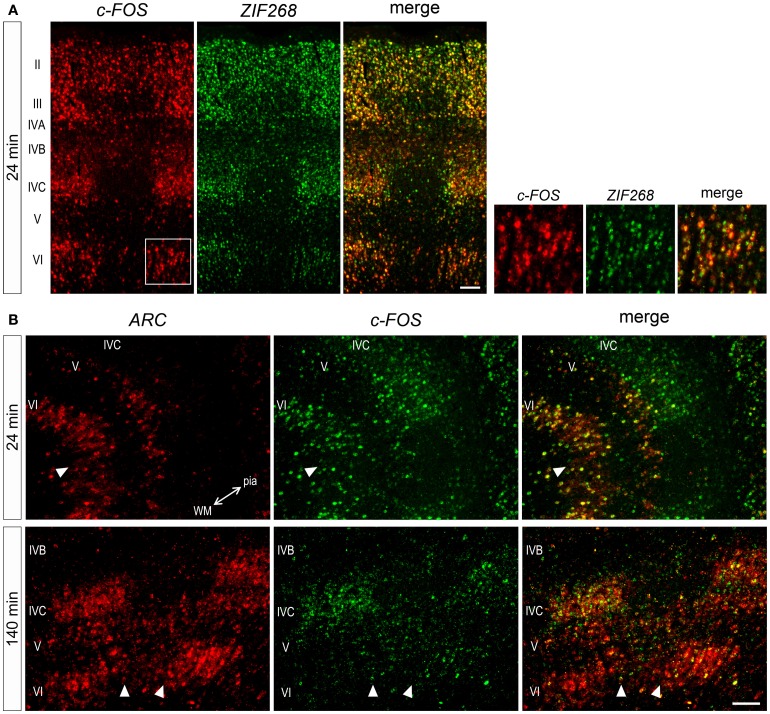
**Double ISH of IEGs in V1 of monocularly stimulated marmosets. (A)** Double ISH of *c-FOS* (red, left) and *ZIF268* (green, middle) mRNAs 24 min after light stimulation. *c-FOS* and *ZIF268* mRNAs were coexpressed in most neurons throughout layers. **(B)** Double ISH of *ARC* (red, left) and *c-FOS* (green, middle) mRNAs in V1. *ARC* and *c-FOS* mRNAs were coexpressed in most neurons in the layers when they were expressed at each time point. The two-way arrow indicates the direction of lamination from pial (pia) to white matters (WM). Arrowheads indicate the boundaries of ODCs. Scale bars: 100 μm.

Considering gene-specific differences of mRNA expressions, we wanted to know whether these mRNAs are expressed within the same or different neurons. To examine this, we carried out double ISH of these three IEG mRNAs. At 24 min, *ZIF268* and *c-FOS* mRNAs were coexpressed in almost all neurons (Figure 7A), indicating that these IEGs might be induced by a similar regulatory mechanism. On the other hand, induction of *ARC* and *c-FOS* mRNAs was not always similar in all layers. For example, at 24 min, both mRNAs were coexpressed in almost neurons in layer VI, although *ARC* mRNA was not yet expressed in layer IVC (Figure 7B). At 140 min, however, *ARC* and c-*FOS* mRNAs were mostly coexpressed in layer IVC (Figure 7B), suggesting that these IEGs were induced in the same neurons, but the timing of *ARC* induction was different between layers IVC and VI. Collectively, these data indicate that parallel transcriptional regulations of these three IEGs take place in the same neuron upon the same stimulation in the marmoset primary visual cortex.

**Figure 8:**
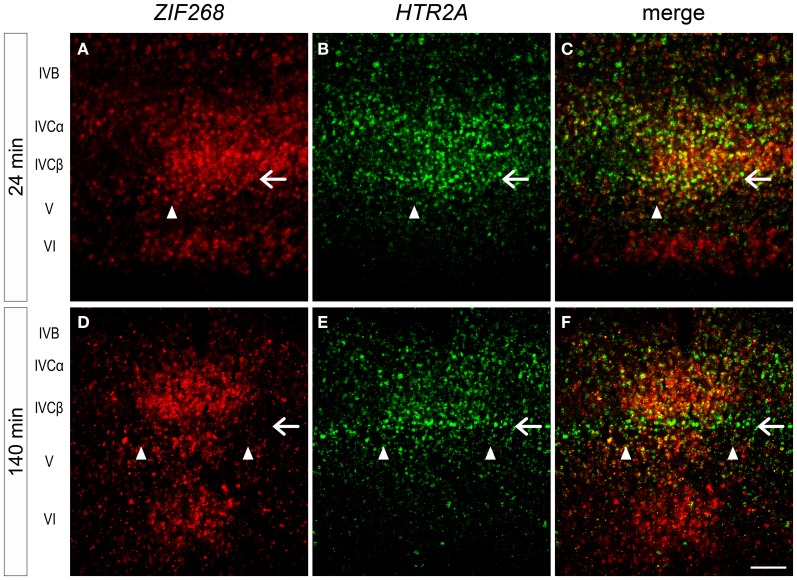
**Double ISH of *ZIF268* and *HTR2A* in monocularly stimulated marmoset V1.** *ZIF268* **(A)** and *HTR2A* **(B)** mRNAs were coexpressed at the border between layers IVCβ and V in the active-eye columns 24 min after light induction (white arrow). **(C)** Merged figure of **(A)** and **(B)**. However, 140 min later, *ZIF268* **(D)** and not *HTR2A* **(E)** mRNA disappeared from this sublayer (white arrow). **(F)** Merged figure of **(D)** and **(E)**. Arrowheads indicate the boundaries of ODCs. Scale bar: 100 μm.

In this series of monocular visual stimulation experiments, we were able to reveal the existence of a sublayer of layer IV that exhibits a unique pattern of gene expression. A previous study showed the narrow (thin) pale-staining rim below the strong ZIF268 signals in layer IVC in marmoset V1 (Markstahler et al., 1998). From its position, this sublayer is considered as equivalent to the blank sublayer of *ZIF268* mRNA observed at 140 min after light induction (showed by the filled star in Figure 5D). However, the *ZIF268* ISH at 24 min did not show such a blank layer at the equivalent lamina position (showed by the filled star in Figure 5C). As described above, *HTR2A* also exhibited a narrow sublayer around this lamina position (Figures 3, 4). To identify this narrow sublayer in more detail, we performed double ISH of *ZIF268* and *HTR2A* mRNAs in the cortical sections of 24- and 140-min-stimulated marmosets (Figure 8). We found that *ZIF268* mRNA lacked the expression in the same sublayer where *HTR2A* mRNA was consistently expressed at 140 min (arrow, Figures 8D–F), while they were coexpressed at 24 min (arrow, Figures 8A–C). Interestingly, *ARC* mRNA expression was absent in this sublayer regardless of the time points (filled star, Figures 5H–J), while *c-FOS* mRNA was expressed uniformly over the entire layer IVC (i.e., layers IVCα, IVCβ, and this thin sublayer) (Figures 5M,N). These expression patterns clearly indicate that there are heterogeneity of the regulation of the activity-dependent gene expression among the IEGs and the following gene expression in marmoset V1.

## Discussion

### Summary of the results

In this study, we carried out a series of monocular visual stimulation experiments using adult marmosets to investigate the time course of a group of activity-dependent genes in V1. The first conclusion from the series of experiments was that all activity-dependent genes clearly revealed the ocular dominance type of expression pattern in monocular stimulated marmoset V1. Second, the spontaneous retinal activity induced a certain level of *HTR1B* and *HTR2A* mRNA expressions without light stimulation. Third, we found that the expressions of the genes examined were regulated in layer- and sublayer-specific manner in the marmoset V1. Each IEG has unique time course of its expression in neurons of each layer in response to visual inputs and is coordinately but differently regulated in the same neuron. These findings illustrate the fine control mechanism of activity-dependent gene regulation in marmoset V1. To the best of our knowledge, this is the first study to investigate a very rapid change of gene expression upon visual inputs in non-human primate V1.

### Functional ocular dominance columns in adult marmosets

The existence of ODCs in adult marmosets has been controversial. It had been believed that marmosets have anatomical ODCs only transiently in their youth (Spatz, 1979, 1989). However, Sengpiel et al. (1996) suggested that ODCs could be observed in adulthood by monocular eyelid suture during development. Chappert-Piquemal et al. (2001) reported the anatomical ODCs in two out of four normal adult marmosets. As we mentioned in the Introduction, “Physiological ODCs” that are termed by these authors have been visualized with ZIF268 immunostaining (Markstahler et al., 1998). On the other hand, most cells in the marmoset V1 showed equal responsiveness through the two eyes by electrophysiological unit recording (Sengpiel et al., 1996). Optical imaging study also failed to detect reliable ODCs in adult marmosets (Roe et al., 2005; McLoughlin and Schiessl, 2006; Valverde Salzmann et al., 2012), despite that the existence of orientation columns (Roe et al., 2005; McLoughlin and Schiessl, 2006) and color domains (Valverde Salzmann et al., 2012) are demonstrated in adult marmoset by this method.

Although previous results suggest the existence of adult ODCs, the number of marmosets used to show ODCs are limited and ODCs are not always observed in adult marmosets as above described. In our study, all adult marmosets (11 in total) that received monocular TTX injection revealed clear ODCs by ISH for activity-dependent genes throughout layers II–VI (Figures 2, 5). We consider it important that the effect of TTX on mRNA expression of IEGs was not only restricted to layer IVC but also observed in all layers. This result suggests that most neurons in layers II–VI of marmoset V1 have ocular dominance along with columnar units as shown in other primates, and these ODCs were detectable by ISH of activity-dependent genes. Our finding is the first report showing structural segregation of the right and the left eye inputs outside layer IVC in marmoset V1, although previous reports using activity-dependent *ZIF268* expression show ODCs in the capuchin monkey (*Cebus*) other than layer IVC (Silveira et al., 1996; Soares et al., 2005). The difference of IEG expression level between active- and inactive-eye columns outside layer IVCβ, however, appeared to be less conspicuous than that of the Old World monkey (Chaudhuri and Cynader, 1993; Chaudhuri et al., 1995; Takahata et al., 2009). This indicates the lesser extent of left and right eye dominance in V1 of marmosets than that of Old World monkeys, which may be a reason why ODCs have not been detected consistently. We also consider that higher sensitivity of ISH method, as compared to ZIF268 immunostaining (Markstahler et al., 1998) may enable to detect ODCs outside layer IVCβ in all monocular deprived marmosets. In general, we could obtain better contrast of pictures by ISH than that by immunostaining (see Figure 5 and also Van der Gucht et al., 2007).

Since the discovery of V1 physiology including “ocular dominance” (Hubel and Wiesel, 1962), the functional ocular dominance is observed through mammals and the study has been targeted in the mouse as a model system to further dissecting the underlying molecular mechanisms (Hensch, 2005). However, no anatomical separation of the right and left LGN projection has been found in the mouse, rat or squirrel and partial separation into vertical cortical stripes in the cat. In primates, prosimian Galago and the representative Old World monkeys show clear anatomical ODCs (see the introductory summary by Spatz, 1979).

There has been controversy about ODCs of New World monkeys including marmosets, because most of them show little or weak anatomical separation of the projections from LGN (see Spatz, 1979; Livingstone, 1996). In spider monkeys (*Ateles alter*), however, Florence et al. (1986) demonstrated clear ODCs in layer IV, which casts an interesting question in regard to the phylogenic significance of ODCs in the New World monkey. Besides the marmoset (*Callithrix*), ODCs in adult animals were observed in the capuchin monkey (*Cebus*) (Silveira et al., 1996), the squirrel monkey (*Saimiri*) (Horton and Hocking, 1996), and the owl monkey (*Aotus*) (Rowe et al., 1978; Kaskan et al., 2007), whereas no ocular segregation was observed in the Saki monkey (*Pitheeia*) (Spatz, 1979; Florence et al., 1986; Livingstone, 1996). In addition our present study, we have recently reported ODCs in owl monkeys by monocular inactivation and gene expression study (Takahata et al., 2012). In this regard, the New World monkeys that have anatomical ocular separations (ODCs) in V1 may be indeed a major group. Although the rich and diverse environment where New World monkeys live have given rise to a quite variation of the visual systems, the ocular segregation in V1 may have evolved in the monkey visual system throughout the phylogeny of prosimians, New World monkeys, and Old World Monkeys. However, it remains for further future studies to test this notion including exceptional species.

### The expression property of *HTR1B* and *HTR2A* genes in marmosetV1 suggests a critical role of spontaneous activity

We previously showed that *HTR1B* and *HTR2A* genes decreased their mRNA levels within 3 h of monocular inactivation by TTX in macaques (Watakabe et al., 2009). Our present data demonstrated the importance of spontaneous retinal activity over visual stimulation in the expression of these mRNAs in V1 (Figure 3). From this result, we suggest that the spontaneous retinal activity-dependent regulation of *HTR1B* and *HTR2A* mRNAs may be a mechanism to ensure a certain level of their expressions in the thalamorecipient neurons in V1, regardless of visual stimulation. By *in vivo* electrophysiological experiments using specific agonist and antagonist for HTR1B and HTR2A receptor proteins, we previously reported that HTR1B and HTR2A exert modulatory effects in macaque V1, increase of S/N ratio and gain control, respectively (Watakabe et al., 2009). Considering our present data, both receptors may always be present in V1 at a certain level, independent of the visual environment, to play these roles in macaque and marmoset V1. Xiang and Prince (2003) report the role of HTR3 receptors and HTR1A receptors of layer V pyramidal cells in rat visual cortex. The specific agonists for HTR3 and HTR1A cause opposite effects for spontaneous IPSC of pyramidal cells by controlling inward and outward currents of interneurons. Therefore, the combination of different subtypes of 5HT receptors may play similar roles in modulating the activity of excitatory neurons. However, it should be noted that *HTR1B* and *HTR2A* are expressed in excitatory neurons and their expressions are regulated in activity dependent manners (Watakabe et al., 2009), which features may have been added during the course of the evolution of the primate visual cortex.

We also note that the light-induced expression of *HTR1B* mRNA in V1 gradually increased upon light stimulation. This observation indicates that two different kinds of activities may control *HTR1B* mRNA. One is the spontaneous retinal activity and the other is the visual stimulation. Considering the rapid decreases upon TTX injection within 3 h (Watakabe et al., 2009), the offset of transcription for *HTR1B* and *HTR2A* is rapidly responded to a blocking spontaneous retinal activity. On the other hand, the slow induction of *HTR1B* mRNA by visual stimulation suggests that the response may be following to the expression of rapidly synthesized transcriptional factors including IEGs upon light induction. Therefore, our results strongly suggest that there are different transcriptional controls of *HTR1B* and *HTR2A* from those of IEGs. However, what kinds of molecules are involved in the activity-dependent gene expression of *HTR1B* and *HTR2A* remains to be revealed and we are currently studying for it.

### Layer-specific regulation of activity-dependent gene expression in marmoset V1

Our series of light induction experiments revealed a fine layer-specific regulation of each activity-dependent gene in marmoset V1 (Figures 4–8). Previous study suggests that Zif268 and JunD immunoreactivities exhibit different laminar patterns in the primate visual cortices under the normal reared condition (Okuno et al., 1997). Our results now revealed the evidence for multiple layer-specific regulation mechanisms in each gene evoked by the visual input in marmoset V1.

Several studies report the time course of IEG expression upon light exposure after dark-adaptation in V1. These studies, however, did not report differential time course for each layer. For example, investigation of c-Fos and Zif268 (mRNAs and immunostains) in rodents revealed rapid induction (within 30 min) of IEGs upon light stimulation, consistent with our finding (Worley et al., 1991; Zangenehpour and Chaudhuri, 2002). Kaplan et al. (1996) reported that 1 or 4 h of light exposure induced both ZIF268 and c-FOS immunostaining in adult cat V1. However no layer difference was reported for either of these cases. In primates, 2 and 5 h light induction experiments have been performed for vervet monkeys (Chaudhuri and Cynader, 1993; Chaudhuri et al., 1995). The authors did not mention the changes of laminar distribution of ZIF268 immunoreactivity, it may be because the laminar expression pattern of ZIF268 changes little after 2 h.

Two aspects of laminar differentiation should be considered to understand the activity-dependent profile in marmoset V1. First, there are highly layer-specific input-output pathways in primate V1. The inputs from parvo- and magnocellular layers of the LGN mainly enter layers IVCα and IVCβ of V1, as well as layers IVA, and VI (Nassi and Callaway, 2009). *HTR1B* was strongly expressed in layer IVC with a greater expression of IVCβ and subjected to spontaneous activity and light stimulation (Figure 3). This pattern of expression seems to well fit to the function of HTR1B receptors in the primary visual cortex in primates (Watakabe et al., 2009). In contrary, other activity-dependent genes (*c-FOS, ZIF268, ARC*, and *HTR2A*) were expressed most of the layers, which suggests their multiple roles in marmoset V1. V1 also receives inputs from the pulvinar nucleus in layer I (Shipp, 2003; Callaway, 2004) or feedback projections from V2 in layer I (Rockland and Pandya, 1979). There is very little expression of all the activity-dependent genes examined in layer I. *OCC1/FSTL1* which we first reported as the visual area-selective and activity dependent gene (Tochitani et al., 2001) is expressed at a significant level in the lower layer III in V2 that probably receives the pulvnar projection (Levitt et al., 1995). This difference of the expression within the pulvinar receiving neurons may be because thalamocortical fibers in layer I contact branches of the apical dendritic bouquets of deeper neurons (Nieuwenhuys, 1994) and in layer I there may be no soma of the neurons that receive the thalamocortical fibers. Second, cell types that constitute each layer in primate V1 are heterogeneous (Thomson and Lamy, 2007). It is quite possible that the neurons in different layers possess different sets of regulatory factors for activity-dependent gene expression. For example, the lack of *ARC* mRNA expression in layer IVC but not in other layers for the initial 24 min suggests that different signaling cascades and transcriptional factors are involved in these neurons. Since different types of neurons coexist in each layer (Thomson and Lamy, 2007). There might be some neural mechanisms to orchestrate activity-dependent transcription among different subtypes of neurons.

In this regard, the sublayer of very bottom part of layer IVC was intriguing, in that *ZIF268, HTR2A*, and *ARC* mRNAs exhibited differential responses to visual stimulation (Figures 4, 5, 8). Presence of thin sublayer of Zif268 mRNA and protein at the same sublamina position has also been observed in vervet monkeys (Chaudhuri and Cynader, 1993; Chaudhuri et al., 1995), and in macaque monkeys (Takahata et al., 2008, 2009). These studies raise the possibility that gene-specific and layer-specific responses to visual stimulation may be conserved among certain primates.

Finally, our results that showed the different temporal and spatial regulation of IEGs, *HTR1B*, and *HTR2A* in marmoset V1 upon the change of retinal inputs suggest that precise cell- and layer-specific transcriptional control of activity-dependent genes is likely to contribute to differential roles of neurons of each layer in visual processing, which should play roles in the formation and maintenance of the highly stratified visual cortex of primates, and thereby to their visual functions.

### Conflict of interest statement

The authors declare that the research was conducted in the absence of any commercial or financial relationships that could be construed as a potential conflict of interest.
